# Floral Scents of a Deceptive Plant Are Hyperdiverse and Under Population-Specific Phenotypic Selection

**DOI:** 10.3389/fpls.2021.719092

**Published:** 2021-09-24

**Authors:** Eva Gfrerer, Danae Laina, Marc Gibernau, Roman Fuchs, Martin Happ, Till Tolasch, Wolfgang Trutschnig, Anja C. Hörger, Hans Peter Comes, Stefan Dötterl

**Affiliations:** ^1^Department of Biosciences, Paris Lodron University of Salzburg, Salzburg, Austria; ^2^Laboratory of Sciences for the Environment, CNRS – University of Corsica, Ajaccio, France; ^3^Lab for Intelligent Data Analytics Salzburg, Paris Lodron University of Salzburg, Salzburg, Austria; ^4^FG Tierökologie, University of Hohenheim, Stuttgart, Germany; ^5^Department of Mathematics, Paris Lodron University of Salzburg, Salzburg, Austria

**Keywords:** *Arum maculatum*, brood-site deception, chemical ecology, geographic variation, hyperdiverse floral scents, phenotypic selection, Psychodidae

## Abstract

Floral scent is a key mediator in plant-pollinator interactions. However, little is known to what extent intraspecific scent variation is shaped by phenotypic selection, with no information yet in deceptive plants. In this study, we collected inflorescence scent and fruit set of the deceptive moth fly-pollinated *Arum maculatum* L. (Araceae) from six populations north vs. five populations south of the Alps, accumulating to 233 samples in total, and tested for differences in scent, fruit set, and phenotypic selection on scent across this geographic barrier. We recorded 289 scent compounds, the highest number so far reported in a single plant species. Most of the compounds occurred both north and south of the Alps; however, plants of the different regions emitted different absolute and relative amounts of scent. Fruit set was higher north than south of the Alps, and some, but not all differences in scent could be explained by differential phenotypic selection in northern vs. southern populations. This study is the first to provide evidence that floral scents of a deceptive plant are under phenotypic selection and that phenotypic selection is involved in shaping geographic patterns of floral scent in such plants. The hyperdiverse scent of *A. maculatum* might result from the imitation of various brood substrates of its pollinators.

## Introduction

Approximately 88% of angiosperms are cross-pollinated by animals (Ollerton et al., 2011) that are attracted to flowers by multifaceted cues (Chittka and Thomson, 2001). Together with visual cues, the main attractant for pollinators is the floral scent (Knudsen et al., 2006; Raguso, 2008). Therefore, the scent has strong effects on pollinator visitation and frequency and, hence, the reproductive success of the plant (Raguso, 2008; Delle-Vedove et al., 2017). With more than 2,000 floral volatile organic compounds (VOCs) described (Knudsen et al., 2006; El-Sayed, 2019), and an average of 20–60 VOCs per species (Knudsen and Gershenzon, 2020), floral scent blends can tremendously vary among species in terms of composition and quantity. Consequently, they facilitate discrimination by pollinators among host plant species and contribute to reproductive isolation of closely related species (Stökl et al., 2009; Friberg et al., 2014).

In addition to interspecific variation, floral scent is also known to vary intraspecifically, both within and among populations (Delle-Vedove et al., 2017). Such intraspecific variability might result directly from abiotic (e.g., temperature; Farré-Armengol et al., 2014) and/or biotic (e.g., herbivores; Kessler and Halitschke, 2009) factors. Given that scent is heritable (e.g., Zu et al., 2016), intraspecific differences can also result from varying evolutionary forces, such as natural selection and genetic drift (Herrera et al., 2006; Majetic et al., 2009). Although not explicitly demonstrated, genetic drift was suggested to be responsible for strong inter-population differences in floral scents (Delle-Vedove et al., 2017) or to counteract pollinator-mediated selection (in two *Yucca* species; Svensson et al., 2006). In contrast, natural selection on floral scent emission, both on total scent amount and individual scent components, has been shown by analyses of phenotypic selection, correlating scent phenotypes and fitness measures (e.g., Parachnowitsch et al., 2012; Gross et al., 2016; Chapurlat et al., 2019).

Phenotypic selection on floral scent can vary intraspecifically, potentially leading to variable adaptive responses to spatially variable pollinator assemblages (Gross et al., 2016). Until now, studies examining phenotypic selection on floral scents have been conducted in rewarding but not in deceptive species, although the latter also often rely on luring and deceiving their pollinators with scents (Jürgens et al., 2013; Schiestl and Johnson, 2013). Compared with their rewarding relatives, non-rewarding species often display higher variation in scent and other traits attractive to pollinators (e.g., Ackerman et al., 2011; Dormont et al., 2014). Furthermore, non-rewarding species are frequently more pollen-limited, e.g., Tremblay et al. (2005). In consequence, they might experience stronger selection on floral scent than rewarding species, as shown for floral traits other than scent (Sletvold and Ågren, 2014).

An ideal target for studying phenotypic selection on scent is the moth fly-pollinated and brood-site deceptive *Arum maculatum* L. (Araceae). This strongly scented (e.g., Kite, 1995; Chartier et al., 2013) plant species attracts its pollinators by olfactory deception (e.g., Kite et al., 1998), shows high variation in fruit and seed sets within and among populations (e.g., Ollerton and Diaz, 1999), and has a geographically variable pollinator spectrum (Espíndola et al., 2011). This perennial herb is widespread in Europe, and the main pollinators are two moth flies, namely *Psychoda phalaenoides* L. and *P. grisescens* Tonn. (Psychodidae). In Central and much of Western Europe, high abundances of female *P. phalaenoides* were found (Espíndola et al., 2011). In other regions, such as Mediterranean Europe and Western France, *A. maculatum* was generally visited by a higher diversity of Diptera (psychodids and non-psychodids) but in much lower abundances, often dominantly by both sexes of *P. grisescens* and not by *P. phalaenoides* (Espíndola et al., 2011). This geographic pollinator variation is particularly pronounced north vs. south of the Alps (Espíndola et al., 2011; Laina et al., unpublished data) and matches a genetic subdivision (amplified fragment length polymorphisms; AFLP) of *A. maculatum* across this geographic barrier (Espíndola and Alvarez, 2011). *Arum maculatum* occurs only up to the submontane level, thus being absent in the Central Alps (Eggenberg et al., 2018). The insects are attracted by the strong dung-like inflorescence scent and not by visual cues of *A. maculatum* (Gfrerer et al., unpublished data) while looking for oviposition sites and/or mating partners (Kite et al., 1998; Espíndola and Alvarez, 2011). Previous analyses have shown that the scent profile of *A. maculatum* consists of up to 60 compounds, also differing among populations in their composition (Diaz and Kite, 2002; Chartier et al., 2013; Marotz-Clausen et al., 2018; Szenteczki et al., 2021; and references therein). At least in part, this scent variation appears to reflect the population variation in pollinator assemblages of *A. maculatum* across its distribution range (Chartier et al., 2013; Szenteczki et al., 2021). However, it is presently unclear whether the pollinator and genetic differences of *A. maculatum* north vs. south of the Alps are also reflected in the species' scent patterns. Nonetheless, it is known that the two main pollinating moth fly species have dissimilar floral scent preferences (Chartier et al., 2013; Szenteczki et al., 2021). Hence, we assume that the dissimilar scent preferences of the two fly species, along with their different floral visitation in regions north vs. south of the Alps, could have led to differing selection pressures on scent among respective regional populations of *A. maculatum* from north vs. south of the Alps.

In this study, we investigated the floral scent characteristics and fruit set (as an indicator for female fitness) of *A. maculatum* in six populations north of the Alps vs. five populations south of the Alps and tested for phenotypic selection on scent in the largest and most extensively sampled population in each of the two regions. Specifically, this study aimed to answer the following: (1) Do scent and fruit sets differ between north vs. south of the Alps, and among populations within regions? (2) Is there phenotypic selection on floral scent? If so, (3) do compounds, under selection differ between northern and southern populations? This study expects to find pronounced population differences in scent both at the inter-regional level and within the southern region, considering the differences in pollinator abundance and diversity between regions and also among southern, but not northern, populations (Espíndola et al., 2011). Additionally, when taking the different olfactory preferences of pollinator species into account, we expect lower fruit set south than north of the Alps, and different signs of selection in the most extensively sampled northern and southern populations.

## Materials and Methods

### Study Species and Populations

Brood-site deceptive *A. maculatum* is a rhizomatous perennial woodland herb (2*n* = 4x = 56) that is widespread throughout Western and Central Europe, including the British Isles, and reaches as far south as Italy, Northern Spain, and the Balkans (Boyce, 2006; Espíndola et al., 2010). It is thermogenically active, exhibits a sapromyiophilous pollination strategy, and emits a strong dung-like scent for attracting moth fly pollinators during the evening on the first day of anthesis (Kite et al., 1998; Marotz-Clausen et al., 2018). The inflorescence of *A. maculatum* consists of a spadix (fleshy spike) and a spathe (bract), is protogynous, and the anthesis lasts <2 days (Lack and Diaz, 1991; Marotz-Clausen et al., 2018). The spathe, which completely encloses the spadix during floral development, partially opens during anthesis to reveal the sterile appendix of the apical part of the spadix. This appendix produces and releases the scent for pollinator attraction, e.g., Lack and Diaz (1991) and Scheven (1994). At the base of the spadix, female (fertile and sterile) flowers are situated lowest, followed upwards by male flowers and staminodes (sterile male flowers). All flowers remain enveloped by the spathe during anthesis, forming a chamber that is closed by the staminodes throughout the female stage to prevent trapped insects from leaving. Pollinators are attracted in the evening on the first day of anthesis, during the female stage, slip and fall into the floral chamber, and are trapped overnight (Lack and Diaz, 1991; Gibernau et al., 2004; Espíndola et al., 2011). On the next morning, during the male stage, they are dusted with pollen, before being released at around noon when the staminodes and spathe wither (Lack and Diaz, 1991; Espíndola and Alvarez, 2011). After pollination in spring, red berry-like fruits develop as an infructescence until summer (Lack and Diaz, 1991).

**Figure 1:**
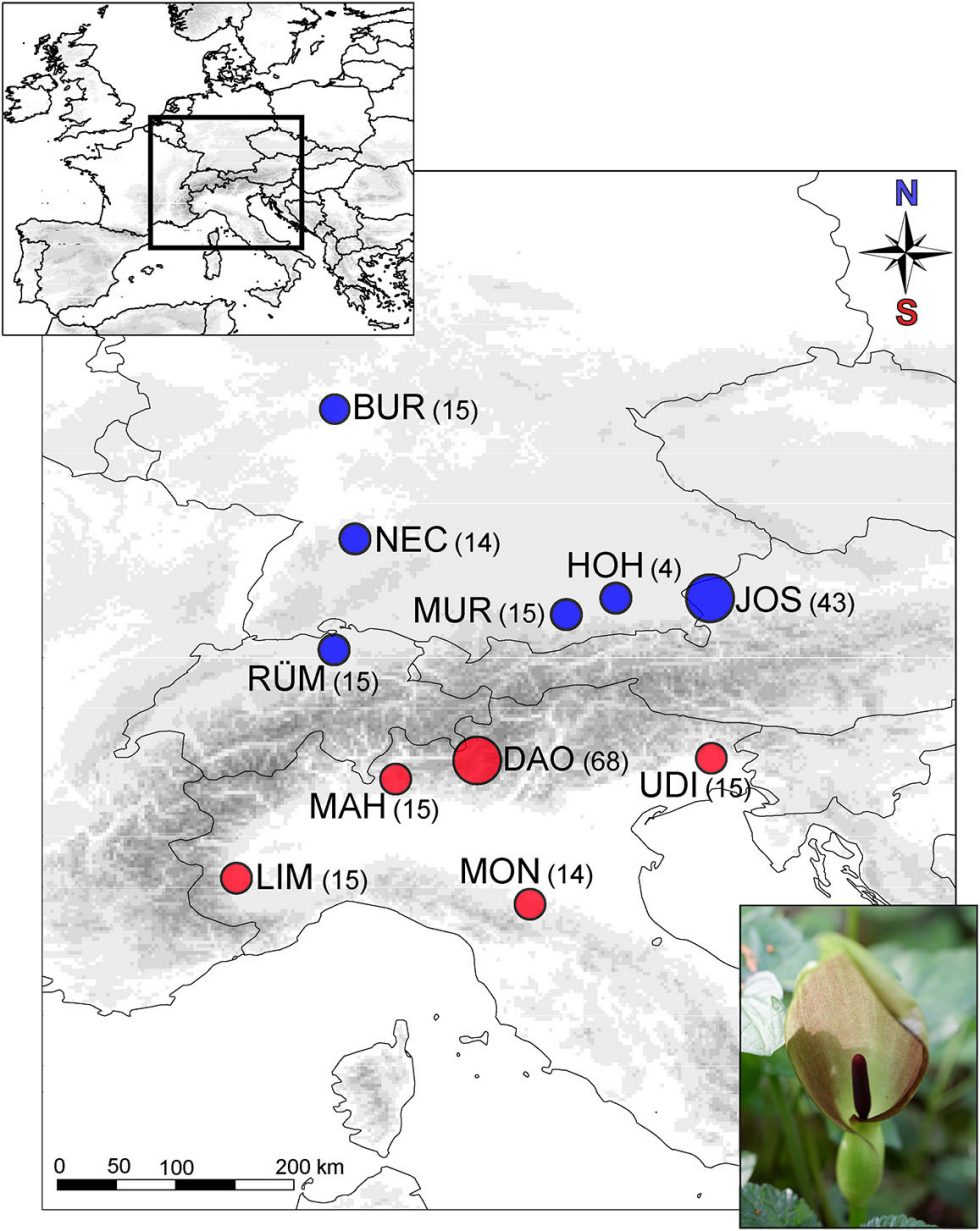
Sampling localities of *Arum maculatum* from the north (blue) vs. south (red) of the Alps. Numbers in brackets give the number of individuals used for scent (and selection) analyses. The two most extensively sampled populations (JOS, DAO) are indicated by larger circles. *North*: JOS, Josefiau; BUR, Burg Hohenstein; HOH, Hohendilching; MUR, Murnau; NEC, Horb am Neckar; RÜM, Rümikon; *South*: DAO, Daone; LIM, Limone-Piemonte; MAH, Santa Maria Hoè; MON, Montese; UDI, Udine.

During springtime in 2017–2019, we collected scents from randomly chosen *A. maculatum* individuals of six populations located north of the Alps (*n* = 106; Northwestern Austria: JOS, Josefiau; Central/Southern Germany: BUR, Burg Hohenstein; HOH, Hohendilching; MUR, Murnau; NEC, Horb am Neckar; Northern Switzerland: RÜM, Rümikon) and five populations from south of the Alps (*n* = 127; Northern Italy: DAO, Daone; LIM, Limone-Piemonte; MAH, Santa Maria Hoé; MON, Montese; UDI, Udine) (Figure 1 and Supplementary Table 1). We kept a minimum distance of 1 m between sampled individuals to avoid sampling potential clones, as *A. maculatum* can also propagate vegetatively by fragmenting rhizomes (Lack and Diaz, 1991). In summer, we harvested fruits from all individuals surveyed for scent. At most sites, we recorded scent and the fruit set of 15 individuals, except for each of the largest population per region (JOS and DAO; *n* = 70 each), and a northern population (HOH; *n* = 7) where only a few individuals had flowered at the time of scent sampling (Figure 1 and Supplementary Table 1).

### Plant Volatile Collection and Analysis

Scent sampling took place on the first day of anthesis during the female stage between 6.00 p.m. and 7:30 p.m. which is the period of maximum scent emission (Marotz-Clausen et al., 2018), employing a non-invasive dynamic headspace technique. We enclosed each inflorescence *in situ* using a plastic oven bag (c. 30 cm × 12 cm; Toppits®, Melitta, Germany) and immediately collected scent for 5 min at 200 ml min^−1^ on adsorbent tubes (inner diameter: 2 mm) filled with a mixture of Tenax-TA (mesh 60–80) and Carbotrap B (mesh 20–40; 1.5 mg each; both Supelco, Germany), using a battery-operated vacuum pump (rotary vane pump G12/01 EB, Gardner Denver Austria GmbH, Vienna, Austria; Marotz-Clausen et al., 2018). In the same way, we collected scent samples from leaves and ambient air as negative controls in each population.

The dynamic headspace samples were analysed by thermal desorption-gas chromatography/mass spectrometry (TD-GC/MS; Marotz-Clausen et al., 2018), and obtained data were handled using *GCMSolution* v.4.41 (Shimadzu Corporation, Kyoto, Japan) (see Supplementary Methods 1 for details). Compounds were chemically identified by comparison of Kováts' retention indices (KRIs), based on commercially available *n*-alkanes (C_7_-C_20_; Sigma Aldrich, Vienna, Austria), and mass spectra to data available in the libraries of Adams (Adams, 2007), FFNSC 2, Wiley9, NIST11, and ESSENTIAL OILS (available in *MassFinder 3*, Hochmuth Scientific Consulting, Hamburg, Germany). We established our own library of mass-spectral and KRIs for semi-automatic analysis (Supplementary Methods 1). Whenever possible, compounds were verified by comparison with authentic reference compounds available in the collection of the Plant Ecology Lab of Salzburg University or with chemically synthesised reference compounds (Supplementary Methods 2). In total, 233 scent samples yielded a sufficiently informative chromatogram and were included in the analyses (Figure 1). Ultimately, a compound was only considered if it occurred in more than three scent samples and did not occur in leaf and air controls.

### Fruit Set

Percentage fruit set, i.e., number of fruits by total number of flowers per individual × 100, was determined as a measure of female reproductive success. For selection analyses, we further estimated relative fruit set, i.e., number of fruits per individual divided by mean number of fruits per given population, (e.g., Gross et al., 2016), for the most extensively sampled populations JOS and DAO. In one southern population (MON), a shallow landslide destroyed all plants, with the exception of one; hence, this population was excluded from fruit set analyses.

### Statistical Analyses

#### Geographic Patterns in Scent and Fruit Set Data

In order to test for geographic differences in floral scent, we performed permutational multivariate ANOVA (permANOVAs; Anderson, 2001) as implemented in the R package *vegan* v.2.6-6 (Oksanen et al., 2019). This was carried out on (1) pairwise Bray–Curtis dissimilarities of either absolute or relative scent data, i.e., the absolute amount of single compounds or the relative amount of single compounds in relation to the total amount of scent in a sample, respectively; (2) pairwise Euclidean distances of both total absolute emission of scent and the total number of floral volatiles per individual. In all these analyses, we used *region* (north vs. south of the Alps) and *population* nested in *region* as explanatory variables (9,999 permutations). Using permANOVA (*population* as an explanatory variable, 9,999 permutations), we also tested for differences in relative and absolute scent between the two most extensively sampled northern (JOS) and southern (DAO) populations by using either (1) all compounds, (2) only those that were under selection and correlated with relative fruit in the *elastic net/Boruta* analyses (see below and Supplementary Methods 3), or (3) those that were not under selection and did not correlate with relative fruit set.

The Bray–Curtis dissimilarity matrices, based on absolute and relative scent data across all populations, were further used to conduct canonical analyses of principal coordinates (CAPs; Anderson and Willis, 2003) with *population* as factor, using the *capscale* function in *vegan* (Oksanen et al., 2019) to visualise similarities and dissimilarities in scent among the samples. For each ordination, we also calculated vectors that represent compounds most correlating with the axes (Pearson correlations with *capscale scores, r* > |0.5|, corrected for false-discovery rate; Benjamini and Hochberg, 1995). Given that CAP is not appropriate to display similarities and dissimilarities in scent between only two populations in a two-dimensional ordination, we used non-metric multidimensional scaling (nDMS) to visualise similarities and dissimilarities in scent among the samples of only JOS and DAO, using only compounds that correlated with relative fruit set or those that did not.

Additionally, we subjected the absolute and relative scent data to random forest analyses (Breiman, 2001) in the R package *randomForest* v.4.6-14 (Liaw and Wiener, 2002) (*ntree* = 9,999 bootstrap samples with *mtry* = 17) to evaluate the distinctness in the scent of northern and southern samples (factor *region*) and among populations within each region (factor *population*). Distinctness was quantified as the average out-of-bag (OOB) error estimate (in %), i.e., the more distinct, the lower the OOB error. From the resulting *randomForest* objects, we further extracted the *importance* measurements to determine volatiles that are critical for regional distinction.

To test for relationships between the dissimilarity of median absolute and relative scent properties of populations and their geographic distances (in kilometres), we performed Mantel tests with the function *mantel* in *vegan* (9,999 permutations, Spearman's rank correlation). To assess whether absolute amounts of single compounds under selection (see below) differ between the two regions, we performed Mann–Whitney *U*-tests. Differences in fruit set across regions and among populations within regions were assessed by an ANOVA (*regions* and *populations* nested within *regions* as factors).

#### Analyses of Phenotypic Selection

To estimate the direction and strength of phenotypic selection on scent compounds, we tested for phenotypic selection (Lande and Arnold, 1983) in the northern JOS and southern DAO populations by correlating relative fruit set with *z*-transformed scent data (standardised to mean = 0, sd = 1; e.g., Parachnowitsch et al., 2012; Gross et al., 2016). These two populations cover a large part of their respective regional scent variation (see Results). As a major challenge, our dataset had a considerably higher number of factors (VOCs) than samples. Previous studies solved this by pre-selecting variables to reduce high dimensionality (e.g., Parachnowitsch et al., 2012) and performed selection analyses only on the most abundant compounds (Knauer and Schiestl, 2017), on principal component scores (e.g., Gross et al., 2016), or physiologically active volatiles (Chapurlat et al., 2019). Due to the very limited knowledge of attractive compounds in the study system (Scheven, 1994; Kite et al., 1998), the fact that the assumptions for principal component analysis were violated, and that also minor volatiles can be under selection (Chapurlat et al., 2019), these solutions were not suitable for our dataset. Instead, we pre-selected volatiles that correlated with relative fruit set *via* an elastic net, i.e., a penalised multivariate linear regression (Zou and Hastie, 2005), and *via* the *Boruta* algorithm (Kursa and Rudnicki, 2010) to identify linear (elastic net) and non-linear (*Boruta*) relationships between total absolute emission and the absolute emission of individual volatiles and relative fruit set (for details see Supplementary Methods 3). Additionally, the scent matrix contained many zeros (non-detects), as many compounds were quite rare (*c*. 70% of VOCs in <50% of samples). This zero-inflation can cause severe problems when fitting linear models, as estimates will be biased (Hogg et al., 2019). In consequence, the influence of an individual scent compound on fruit set can be either overestimated or underestimated, leading to potentially wrong conclusions. To quantify the impact of non-detects on elastic net estimates, we performed a simulation study for JOS and DAO separately before the pre-selective analyses (see Supplementary Methods 3, Supplementary Figure 1). Based on the simulation results, we obtained 93 and 81 scent compounds for JOS and DAO, respectively, each of which were then included in both the elastic net regression and the *Boruta* analyses (Supplementary Methods 3, Supplementary Figure 1). For the JOS population, elastic net and *Boruta* recovered 19 and four volatiles, respectively, whereby the latter were already among the linear ones (see Results). In the southern DAO population, no volatile correlated with fruit set in the elastic net but three in the *Boruta* analysis. None of these volatiles was detected for both populations (see Results). Additionally, the total absolute scent amount did not correlate with the fruit set in any of the analyses.

To ultimately test for phenotypic selection (Supplementary Figure 1), we subjected those volatiles selected by the elastic net model (Supplementary Methods 3) to multivariate linear regression (linear β-gradients; Lande and Arnold, 1983) and subjected those volatiles identified by the *Boruta* analyses (Supplementary Methods 3) to multivariate quadratic regression (non-linear/quadratic γ-gradients; Lande and Arnold, 1983) by squaring the terms and doubling resulting estimates (Stinchcombe et al., 2008). For the multivariate regression model of the southern (DAO) population, we excluded the plant individual “DAO076,” as it was determined by Cook's distance as an outlier influencing the model (*D*_DAO076_ = 235.4). Although elastic net handles multicollinearity well, volatiles identified to correlate with fruit set might still correlate with each other (*L*_2_ penalty, see Supplementary Methods 3). Therefore, we also tested for multicollinearity within the multivariate regression models by calculating the variance inflation factor (VIF) (R package *car* v.3.0.8; Fox and Weisberg, 2019) for each scent compound in each model. For the northern (JOS) model, the VIF values of various compounds were high (>5), while for the unknowns UNK1496 and UNK1503 they even exceeded 10, a threshold that indicates strong multicollinearity (Quinn and Keough, 2002). After including these two compounds as an interaction term, the VIF values of most compounds were <5, except for 3-octanol and UNK1279 (VIF>6). After further including the interaction of the latter two volatiles in the model, the VIF values of all volatiles were <4. Based on this, the final northern (JOS) model had an adjusted *R*^2^ value of 0.71. For the southern (DAO) model, all VIF values were <2 (adjusted *R*^2^ = 0.26). All statistical analyses were performed in R v.4.0.2 (R Core Team, 2020).

## Results

### Floral Scent

The total absolute amount of scent was highly variable among the 233 sampled individuals of *A. maculatum* (range of 1–2,052 ng inflorescence^−1^ h^−1^; Table 1). When taken together, northern plants released a 3-fold lower amount of scent than those from the South, along with differences among populations within regions (permANOVA: *region*: pseudo-*F*_(1, 222)_ = 25.7, *population* nested within the *region*: pseudo-*F*_(9, 222)_ = 5.36, both *P* < 0.001). For three of the five southern populations (MAH, MON, LIM; Figure 1), we estimated a median scent amount of *c*. 200 ng inflorescence^−1^ h^−1^, while DAO and UDI showed 1.5-fold higher and 5-fold lower amounts, respectively (Table 1 and Supplementary Table 1). For three of the six northern populations (MUR, NEC, RÜM; Figure 1), median estimates ranged between 40 and 81 ng inflorescence^−1^ h^−1^, while amounts in the remaining populations were manifold higher (JOS and HOH) or lower (BUR) (Table 1 and Supplementary Table 1).

**Table 1:** Median amounts of total absolute and relative (contribution of single compounds to total scent) inflorescence scent of *Arum maculatum* surveyed in six and five populations north and south of the Alps, respectively.

		**North** ** (*n* = 106)**	**JOS** ** (*n* = 43)**	**BUR** ** (*n* = 15)**	**HOH** ** (*n* = 4)**	**MUR** ** (*n* = 15)**	**NEC** ** (*n* = 14)**	**RÜM** ** (*n* = 15)**	**South** ** (*n* = 127)**	**DAO** ** (*n* = 68)**	**LIM** ** (*n* = 15)**	**MAH** ** (*n* = 15)**	**MON** ** (*n* = 14)**	**UDI** ** (*n* = 15)**
Median total absolute amount of scent trapped (ng inflorescence^−1^ h^−1^)		67.4	167.2	13.0	565.8	80.7	39.4	41.7	214.7	311.4	203.8	196.9	201.4	42.3
Total number of volatiles		285	271	186	195	213	212	216	277	257	210	217	188	184
**KRI/Compound class**	**Compound name**													
Aliphatic components													
893	2-Heptanone^*^	1.4	1.4	2.4	0.8	1.3	1.1	1.4	6.9	9.3	0.3	2.9	11.9	4.0
902	2-Heptanol^*^	0.1	0.1	0.3	0.3	0.2	0.2	0.1	1.2	1.9	tr	0.8	2.5	0.5
982	1-Octen-3-ol^*^	1.9	2.4	tr	2.3	2.0	1.8	1.3	0.3	0.4	tr	6.4	tr	1.3
1,096	2-Nonanone^*^	0.2	0.1	0.1	0.1	0.2	0.1	0.2	0.7	0.9	tr	0.2	1.3	0.4
	23 more aliphatic components <1%	0.5	0.5	0.4	2.0	1.7	1.3	0.5	0.7	0.9	0.9	2.0	1.0	0.7
Aromatic components													
1,076	*p*-Cresol^*^	4.2	1.8	0.1	19.4	11.9	9.2	1.5	0.5	0.5	0.3	0.7	0.6	0.9
	4 more aromatic components <1%	tr	tr	tr	0.6	0.3	0.1	0.1	tr	tr	tr	0.1	tr	tr
C5-branched chain components													
	4 C5-branched chain components <1%	tr	tr	tr	0.1	tr	0.2	0.1	tr	tr	tr	tr	tr	tr
Nitrogen-bearing components													
965	β-Lutidine	0.2	0.1	0.7	0.2	0.4	0.4	0.3	0.1	tr	0.6	0.1	tr	1.3
1,310	Indole^*^	24.2	22.3	20.8	12.6	24.6	33.4	35.6	11.9	11.9	24.8	8.8	12.3	9.5
	5 more nitrogen-bearing components <1%	0.1	0.1	tr	0.1	tr	tr	0.3	0.1	tr	0.1	0.1	tr	tr
Irregular terpenes													
	3 irregular terpenes <1%	tr	tr	0.4	0.3	tr	0.6	0.1	0.1	0.1	0.2	tr	0.3	0.1
Monoterpenoids													
914	3,7-Dimethyloct-1-ene^*^	1.5	1.2	1.1	2.6	2.1	1.8	1.7	4.0	4.3	4.1	2.4	4.6	2.7
935	α-Citronellene^*^^§^	0.4	0.3	0.5	0.9	0.5	0.5	0.4	1.3	1.3	1.9	1.1	1.3	1.0
949	β-Citronellene^*^^§^	4.2	3.5	8.0	11.6	3.4	7.1	3.5	9.7	10.8	10.1	6.5	9.9	8.2
972	3,7-Dimethyloct-2-ene^*^	3.1	1.8	2.8	5.1	9.6	2.6	3.3	4.3	4.5	4.4	5.6	2.1	3.2
982	Sabinene^*^	0.2	tr	1.0	0.2	tr	0.4	0.4	0.4	0.3	1.4	0.3	1.2	tr
1,005	2,6-Dimethylocta-2,6-diene^*^	1.2	0.9	1.0	3.4	4.1	1.0	1.5	1.7	1.8	1.7	1.9	0.5	1.6
1,076	Dihydromyrcenol	tr	tr	tr	tr	tr	tr	tr	0.4	0.4	1.0	tr	0.2	0.5
	21 more monoterpenoids <1%	0.3	0.4	tr	2.2	0.9	0.6	0.9	0.6	0.7	1.9	1.2	0.4	1.1
Sesquiterpenoids													
1,357	Bicycloelemene	0.4	0.5	0.1	0.5	1.9	0.1	0.5	0.2	0.1	0.2	0.6	0.1	0.9
1,399	α-Copaene^*^	1.0	1.8	1.3	0.5	0.5	0.7	0.8	0.8	0.6	1.0	1.0	1.6	0.6
1,434	Isocaryophyllene	0.9	1.3	0.7	0.4	0.6	0.5	1.1	0.9	0.7	1.2	1.3	1.2	0.8
1,450	β-Caryophyllene^*^	3.0	5.5	3.0	2.3	1.4	2.7	2.7	2.9	2.2	3.0	4.0	5.3	2.8
1,484	α-Humulene^*^	2.8	4.7	2.8	1.7	1.2	2.3	2.7	2.3	1.6	2.5	3.0	4.0	2.6
1,501	Germacrene D^*^	0.9	1.3	1.4	0.3	0.3	0.9	0.7	0.5	0.3	0.5	0.7	1.3	0.7
1,520	Bicyclogermacrene	0.9	1.0	tr	0.6	2.1	tr	1.3	0.4	0.2	0.2	1.3	tr	1.7
1,547	δ-Cadinene	1.2	1.9	1.4	0.6	0.6	1.5	1.1	0.4	0.3	0.5	0.7	0.4	1.0
	10 more sesquiterpenoids <1%	0.4	0.5	0.3	0.4	0.3	0.5	0.6	0.3	0.1	0.3	0.4	0.7	0.3
Unknown compounds													
829	UNK 829 *m/z:* 54,67,110,41,81,39	0.3	0.8	tr	0.2	0.2	0.3	0.1	tr	tr	tr	2.0	tr	0.3
1,394	UNK 1394 *m/z:* 69,55,41,82,95	0.2	0.2	tr	0.1	0.6	0.2	0.2	0.1	0.1	0.1	1.1	0.2	0.1
1,409	UNK 1409 *m/z:* 81,55,67,95,41	0.2	0.2	tr	0.4	0.4	0.1	0.1	0.2	0.2	0.1	0.6	0.2	1.1
1,415	UNK 1415 *m/z:* 69, 81,41,95,55	3.7	3.9	1.7	2.3	7.3	3.7	3.4	3.8	3.1	2.8	10.4	2.3	11.3
1,492	UNK 1492 *m/z:* 105,161,91,41,93	1.7	2.7	1.4	0.3	0.5	0.5	1.8	1.2	1.0	1.4	1.6	3.1	0.6
1,503	UNK 1503 *m/z*: 81,107,163	0.8	0.9	0.2	0.4	0.8	0.6	1.0	0.2	0.2	0.1	0.4	0.2	0.3
1,524	UNK 1524 *m/z:* 105,161,204,119,93	0.7	1.0	1.3	0.2	0.2	0.8	0.7	0.4	0.2	0.4	0.5	0.9	0.5
1,699	UNK 1699 *m/z:* 81,163,191,95,123	3.6	4.1	3.0	0.5	1.8	3.2	5.2	1.3	1.1	1.6	2.0	0.8	3.2
	189 more unknowns <1%	2.9	3.9	1.6	4.7	4.9	3.8	4.9	2.7	2.2	4.8	4.6	3.4	3.7

*Volatiles are ordered according to compound class, and within class by Kováts' retention index (KRI). The total number of volatiles is also given.*

* *Identification of compound was verified by authentic standards; tr, trace relative amount (< 0.05%); m/z, mass-to-charge ratio in decreasing order of abundance. North: JOS, Josefiau; BUR, Burg Hohenstein; HOH, Hohendilching; MUR, Murnau; NEC, Horb am Neckar; RÜM, Rümikon; South: DAO, Daone; LIM, Limone-Piemonte; MAH, Santa Maria Hoè; MON, Montese; UDI, Udine.*

§ *Synthetic (+)-α- and (+)-β-Citronellene coeluted with naturally detected α- and β-Citronellene on a chiral column (MEGA-DEX DMT Beta SE, 30 m × 0.25 mm ID, 0.23 μm film) (Gfrerer et al., unpublished data).*

*North and South columns present the regional median of the corresponding populations (following columns). Volatiles with a median amount of <1% in any population are pooled*.

**Figure 2:**
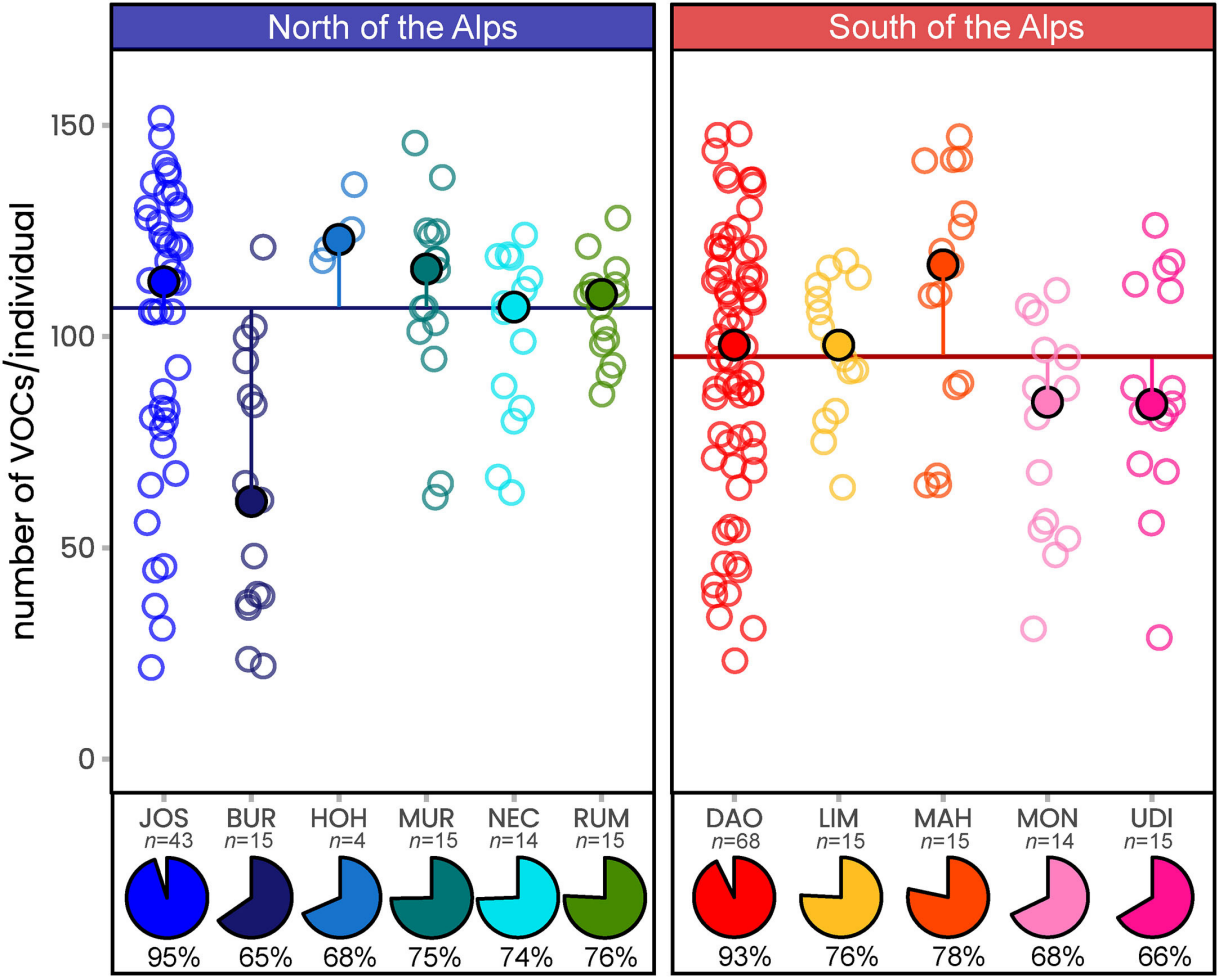
The number of floral scent compounds recorded in *Arum maculatum* individuals from populations north and south of the Alps. Filled circles denote the population median of the number of volatiles per individual; the vertical lines indicate the distance to the region median (horizontal line); open circles mark the number of volatiles detected in the individual samples. Pie charts indicate the percentage of volatiles detected per population (*n*, sample size) compared to the number of compounds detected across all samples (289 compounds). See Figure 1 and Supplementary Table 1 for identification of population codes.

Across all scent samples, we detected a total of 289 floral volatiles (285 north vs. 277 south), of which 92 could be chemically identified (Table 1 and Supplementary Table 2). A median of 102 compounds per individual was recorded (Figure 2), and the number of compounds was independent of the region (permANOVA: pseudo-*F*_(1, 222)_ = 1.98, *P* = 0.16) but varied among populations within regions (pseudo-*F*_(9, 222)_ = 4.57, *P* = 0.001). At the population level, between 186 (BUR) and 271 (JOS) compounds were recorded in the North, and between 188 (MON) and 257 (DAO) in the South (Figure 2). The two most extensively sampled northern (JOS) vs. southern (DAO) populations covered 96 vs. 94% of their respective regional diversity (Figure 2) and together 99% (287/289) of the total number of compounds (Table 1 and Supplementary Table 2). The five most frequent compounds found in more than 99% of the samples were the nitrogen-bearing compound indole, the monoterpenoids 3,7-dimethyloct-1-ene and β-citronellene, the sesquiterpenoid β-caryophyllene, and the unidentified UNK1492 (Table 1).

The absolute amounts of single compounds significantly differed between regions (permANOVA: pseudo-*F*_(1, 222)_ = 22.53, *P* < 0.001), between JOS and DAO only (pseudo-*F*_(1, 109)_ = 9.95, *P* < 0.001), and among populations within regions (pseudo-*F*_(9, 222)_ = 6.44, *P* < 0.001). However, differences were more pronounced between regions than among populations within regions (*north* vs. *south* OOB error: 10.3%; among populations within *north* OOB error*:* 27.3%; within *south* OOB error: 25.2%). Only a few abundant compounds dominated the scent bouquet of *A. maculatum*, including indole, β-citronellene, the unknown UNK1415, and 3,7-dimethyloct-2-ene (all abundant in both regions), *p*-cresol (most abundant only north), and 2-heptanone (only south, Table 1).

We also detected differences in the relative amounts of scent compounds between regions (permANOVA: pseudo-*F*_(1, 222)_ = 30.18, *P* < 0.001), between JOS and DAO only (pseudo-*F*_(1, 109)_ = 22.81, *P* < 0.001), and among populations within regions (pseudo-*F*_(9, 222)_ = 4.90, *P* < 0.001; Figure 2). Again, these differences were more pronounced at the inter-regional than within-region levels (*north* vs. *south* OOB error: 9%; among populations within *north* OOB error*:* 35.8%; among populations within *south* OOB error: 24.4%; see also Supplementary Figure 2).

Across all populations, variation in absolute or relative amounts of scent could not be explained by their geographic distances (Mantel's *Rho* = 0.108, *P* = 0.25 and *Rho* = −0.154, *P* = 0.85, respectively).

**Figure 3:**
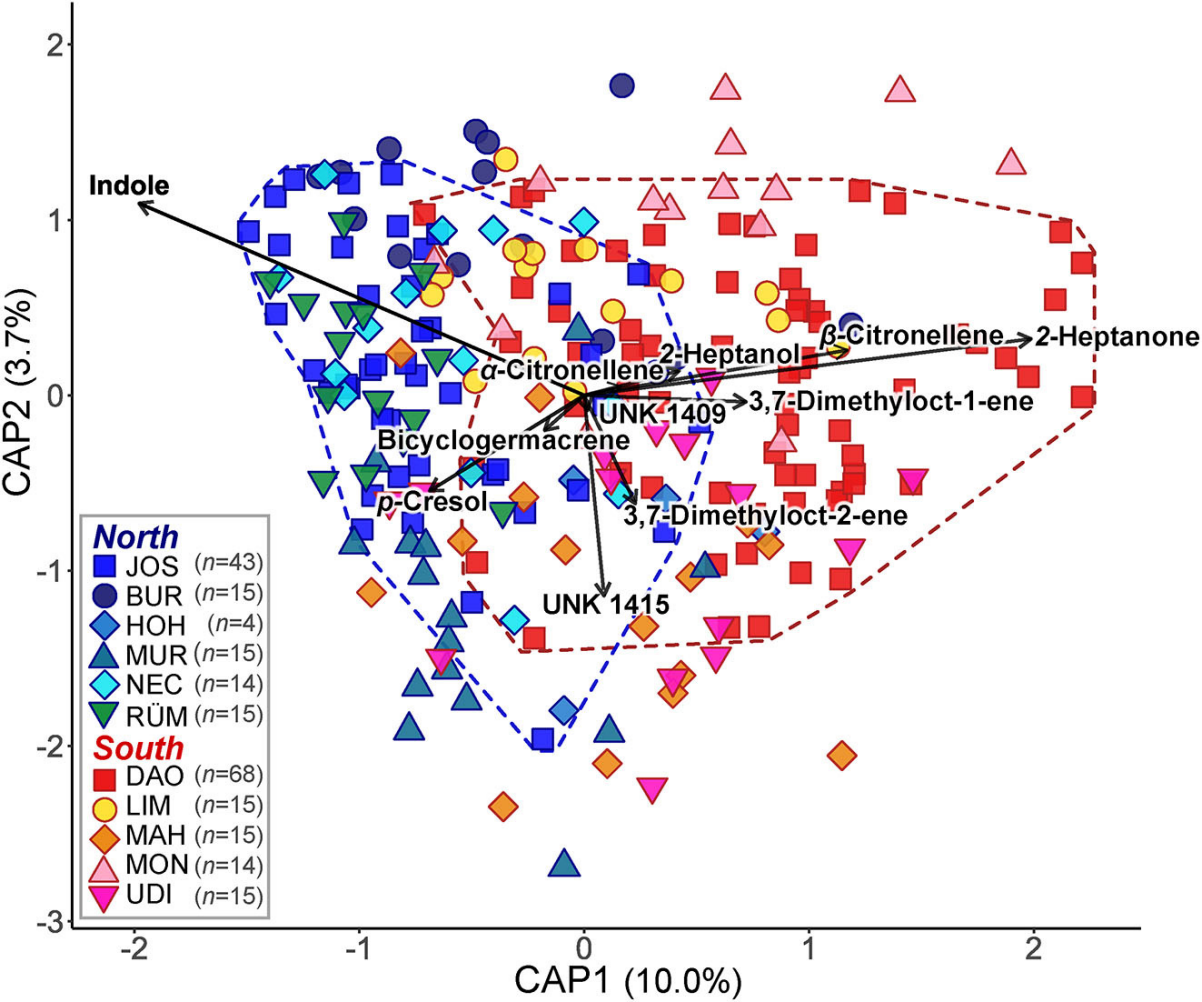
Canonical analysis of principal coordinates (CAP) based on a Bray–Curtis dissimilarity matrix of relative floral scent in *Arum maculatum* individuals from populations north and south of the Alps. *n* denotes the sample size per population. The vectors depict the volatiles most correlating with the *capscale* scores. The coloured dashed lines delineate the individual scent variation of the two most extensively sampled populations JOS (blue) and DAO (red). See Figure 1 and Supplementary Table 1 for identification of population codes.

Among the 25 compounds each that were most responsible for regional differences in the absolute and relative datasets in the *randomForest* analyses, 20 were common to both datasets (Supplementary Table 3). These 20 compounds included 2-heptanone, 2-heptanol, and α- and β-citronellene, all of which were more abundant (in relative and absolute amounts) south of the Alps, and 1-pentadecanol, the unknown UNK1503, *p*-cresol, and indole, which occurred in higher amounts north of the Alps (Table 1 and Supplementary Tables 2, 3). Many of these compounds, and some non-overlapping ones (absolute: α-copaene, β-caryophyllene; relative: UNK1409, bicyclogermacrene), explained most of the variation in scent among all samples (for relative data see Figure 3; for absolute data see Supplementary Table 4).

There was also a considerably high variation in scent within populations, most prominently in the most extensively sampled northern (JOS) and southern (DAO) populations, which harboured almost all of the absolute and relative scent variation of their respective regions (for relative data see Figure 3).

### Fruit Set

**Figure 4:**
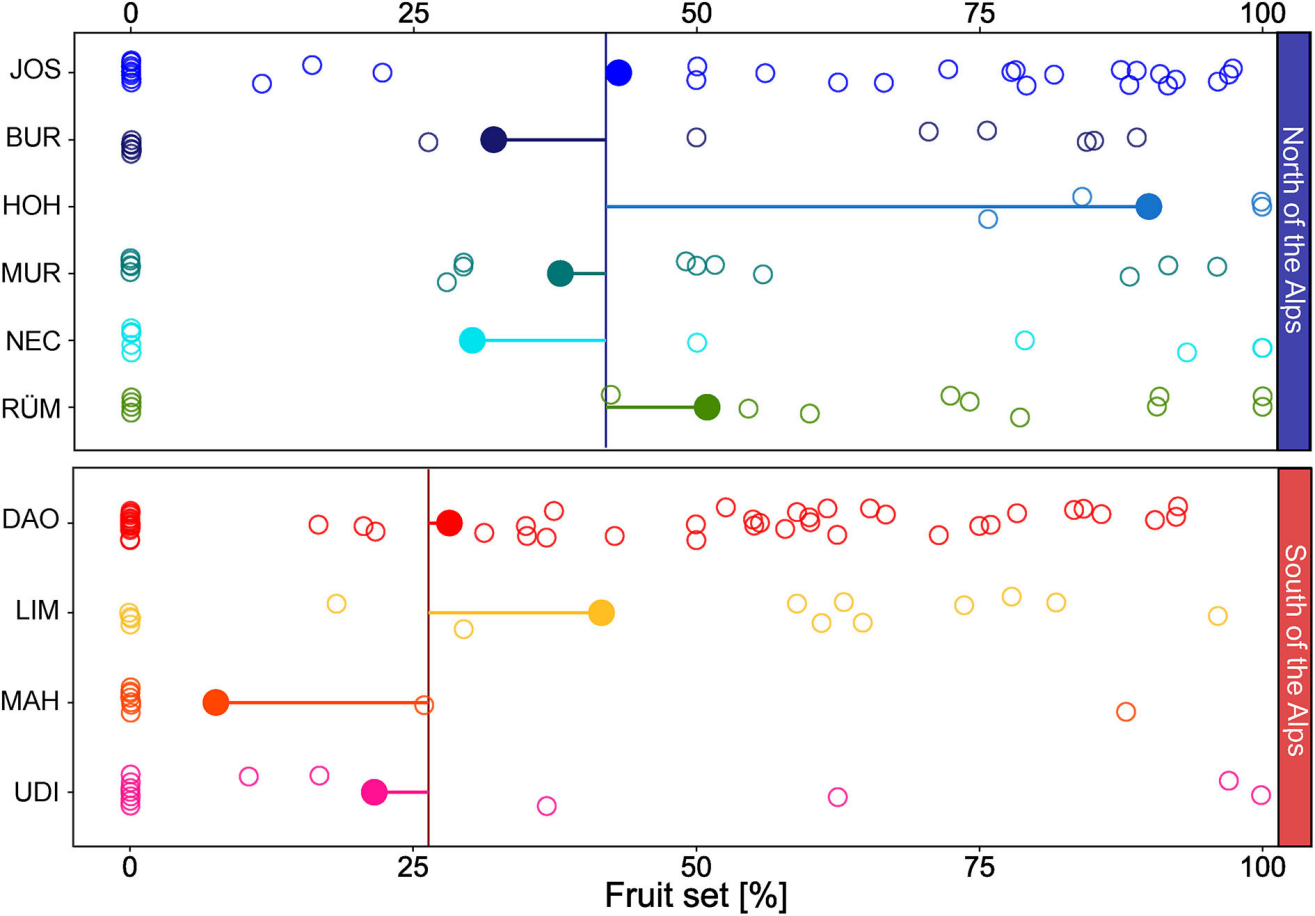
Fruit set (% female flowers that developed into fruits) of *Arum maculatum* individuals from populations north and south of the Alps. Filled circles denote the population mean of the fruit set; horizontal lines indicate the distance to the region mean (vertical line); the open circles mark the fruit set of each individual. See Figure 1 and Supplementary Table 1 for identification of population codes.

Among the 233 individuals surveyed for inflorescence scent, 113 set fruit in summer. Percentages of fruit set were significantly higher north of the Alps (42 ± 41% mean ± *SD*, 0–100% Min–Max) than south of the Alps [26 ± 33% mean ± *SD*, 0–100% Min–Max; Figure 4; *region*: *F*_(1, 209)_ = 10.11, *P* = 0.002] and differed significantly among populations within regions [*population* nested within *region*: *F*_(8, 209)_ = 2.23, *P* = 0.03].

### Phenotypic Selection on Scent

**Figure 5:**
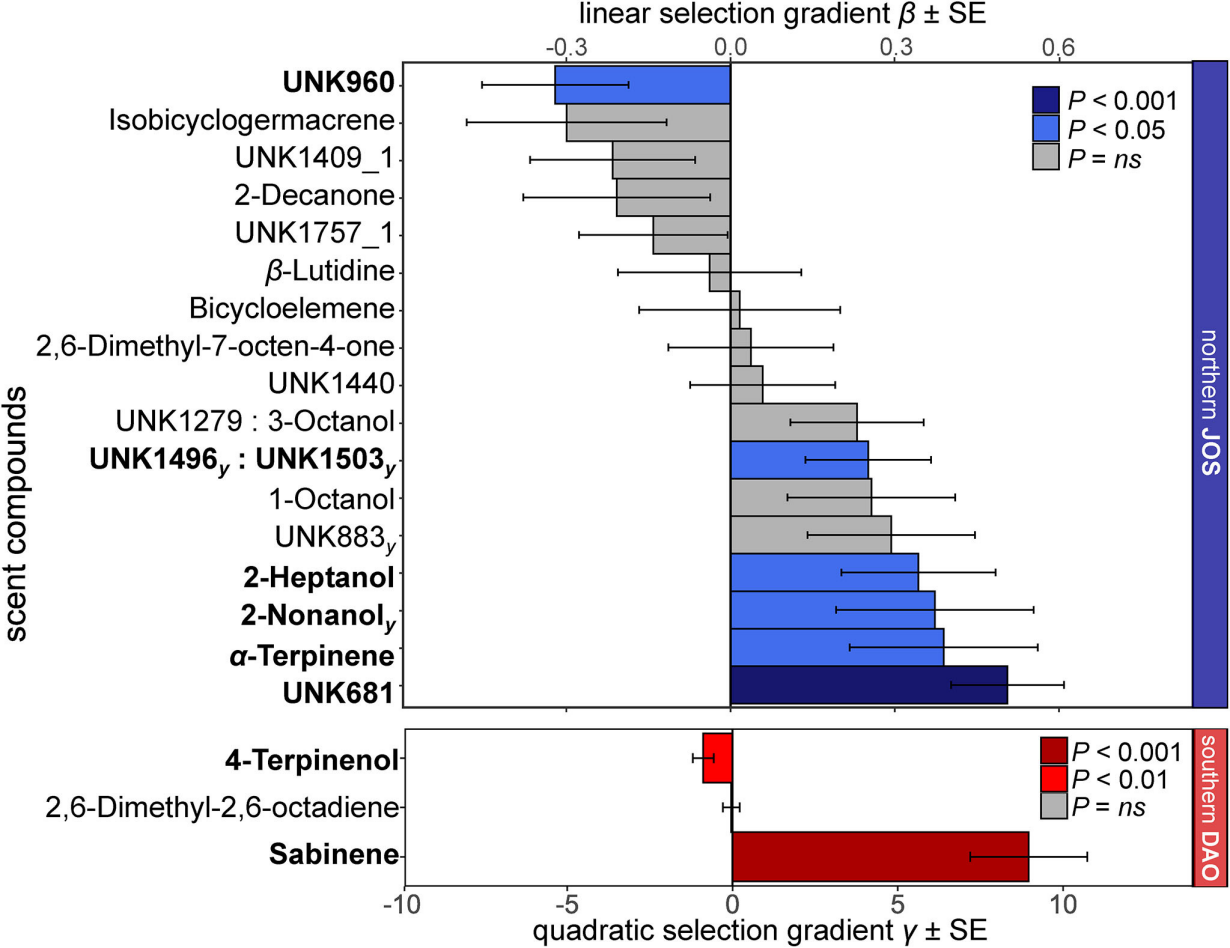
Linear selection gradients β and non-linear quadratic selection gradients γ (and their standard errors, SE) for individual floral scent compounds in the most extensively sampled *Arum maculatum* populations from north (JOS, blue, *n* = 43) and south (DAO, red, *n* = 68) of the Alps. Only compounds that correlated with relative fruit set in the elastic net/*Boruta* analyses are shown (see Material and Methods). Scent compounds under significant selection (*P* < 0.05) are in bold and their bars are coloured. Note the different scaling for linear (β) and non-linear (γ) selection. For the northern population, compounds that were also detected by the non-linear *Boruta* analyses are indicated with a subscript (γ).

In the most extensively sampled northern (JOS) and southern (DAO) populations, we tested 19 and three compounds for phenotypic selection, respectively, as they correlated with relative fruit set in the elastic net and *Boruta* analyses (see section Materials and Methods; Supplementary Methods 3). Among those 22 compounds, seven showed signals of linear phenotypic selection (two of which as an interaction), all in the north, and two for non-linear (quadratic) phenotypic selection, all in the south (Figure 5). Seven of the overall nine compounds that were under phenotypic selection correlated positively with relative fruit set (linear: 2-heptanol, 2-nonanol, α-terpinene, UNK681, and UNK1496 together with UNK1503; non-linear: sabinene), while two correlated negatively (linear: UNK960; non-linear: 4-terpinenol; Figure 5 and Supplementary Table 5).

Among the 25 compounds that most strongly contributed to the absolute (and relative) differences in scent between the regions, only four were under selection (north: 2-heptanol, 2-nonanol, UNK681, UNK1503), but not others (e.g., 2-heptanone, α- and β-citronellene; Supplementary Table 3). Differences in absolute and relative scent traits between the northern JOS and the southern DAO populations remained significant, regardless of performing permANOVA separately on the nine compounds that correlated with relative fruit set in the elastic net/*Boruta* analyses and were under selection [absolute vs. relative datasets: pseudo-*F*_(1, 109)_ = 18.4 vs. 30.8, both *P* < 0.001], or on the 93 compounds that were not under selection and did not correlate with fruit set [absolute vs. relative datasets: pseudo-*F*_(1, 109)_ = 9.8 vs. 24.5, both *P* < 0.001; see Supplementary Figure 3, Supplementary Methods 3].

## Discussion

Our study shows that *A. maculatum* has hyperdiverse inflorescence scents that, in agreement with genetic and pollinator patterns, differ in their composition between populations north vs. south of the Alps. Scent differed not only among southern but also among northern populations, although the pollinator spectrum only differed in populations south of the Alps (Espíndola et al., 2011; Laina et al., unpublished data). As expected, samples from the southern populations had lower fruit set than northern ones, which agrees with lower insect numbers reported in the south (Espíndola et al., 2011; Laina et al., unpublished data), and different signs of phenotypic selection were found in the most extensively sampled northern and southern populations.

### Hyperdiversity of Floral Scent

With 289 floral volatiles recorded, the inflorescence scent diversity of *A. maculatum* is extraordinarily high and not matched by any other plant species to the best of our knowledge. In fact, we are not aware of any species from which more than 200 floral compounds are reported, a number that a single *A. maculatum* individual can reach by three quarters (max. = 152 VOCs; Figure 2). This difference in the number of scent compounds between *A. maculatum* and other species cannot just be explained by differences in techniques used for scent analyses, given that scents of a high number of species were analysed using a similar approach as we did (dynamic headspace and thermal desorption of samples; Gottsberger et al., 2013; Borchsenius et al., 2016; Lukas et al., 2019). Species closest to the high number of VOCs in *A. maculatum* include the sapromyiophilous *Sauromatum guttatum* (Araceae, with altogether 196 different VOCs; Skubatz et al., 1996; Hadacek and Weber, 2002) and *Aristolochia gigantea* (Aristolochiaceae, 168 VOCs; Martin et al., 2017), as well as the insect-pollinated and rewarding *Geonoma macrostachys* (Arecaceae, 176 VOCs; Borchsenius et al., 2016) and *Echinopsis ancistrophora* (Cactaceae, 145 VOCs; Schlumpberger and Raguso, 2008). Other species for which *c*. 100 VOCs are described likewise include insect-pollinated and rewarding species [e.g., *Philodendron bipinnatifidum* (Araceae), Gottsberger et al., 2013; *Pyrus communis* (Rosaceae), Lukas et al., 2019], but also the sexually deceptive orchid *Ophrys sphegodes* (Ayasse et al., 2000). Thus, high numbers of compounds are found across a wide range of plant families and are apparently not restricted to a specific pollination system.

One explanation for the high diversity of scent compounds in *A. maculatum* is that this species likely imitates the various breeding substrates of its moth fly pollinators, all potentially differently scented. The two main pollinators, *P. phalaenoides* and *P. grisescens*, breed in a variety of different substrates such as rotting manure from cattle and horse, fungi (*P. grisescens*), waste pits, mud-flats, plant litter in drainages (*P. phalaenoides*), and the hygropetric zones of river banks and ponds (Satchell, 1947; Ježek, 1990; Sigsgaard et al., 2020). *Arum maculatum* emits compounds described from several substrates, such as cattle and horse manure (*e.g.*, indole, *p*-cresol, skatole), fungi (e.g., 1-octen-3-ol, (*E*)-2-octen-1-ol, 3-octanone), and general degrading and fermenting plant or animal material (e.g., 2,3-heptanedione, acetoin, butanoic acid) (Dormont et al., 2010; Jürgens et al., 2013). Highly specialised deceptive plant systems frequently rely on only a few volatiles to attract pollinators; they seem to imitate a more specific model, thus releasing less complex scent blends (e.g., Wee et al., 2018).

The number of volatiles detected across the 233 samples (11 populations) of *A. maculatum* (289 VOCs) is five to 10 times higher than previously reported for this species (18–61, and 143 VOCs in total; Scheven, 1994; Diaz and Kite, 2002; Chartier et al., 2013; Marotz-Clausen et al., 2018; Szenteczki et al., 2021; and references therein). This discrepancy cannot be explained by differences in sample size, as a similar number of individuals were surveyed in those previous studies (*n* = 222 in total, representing 23 populations). Interestingly, we found a similar number of compounds in some individuals (up to 152 VOCs; *median* of 102; Figure 2) as overall detected previously (Diaz and Kite, 2002; Chartier et al., 2013; Marotz-Clausen et al., 2018; Szenteczki et al., 2021; and references therein). With the exception of two studies (Marotz-Clausen et al., 2018; Szenteczki et al., 2021), each sharing one of our sampled populations (JOS and MON, respectively), all previous studies sampled scents in other populations across Europe (Kite, 1995; Diaz and Kite, 2002; Chartier et al., 2013). Thus, some of the differences in the number of *A. maculatum* compounds detected across studies might reflect population-specific scent characteristics (see Figure 2). However, more importantly we believe that the discrepancy in the number of compounds recorded largely reflects differences in methodology between the present and previous studies. For example, these are: higher sensitivity of modern GC/MS systems, usage of less selective adsorbent agents [Carbotrap/Tenax-TA vs. polydimethylsiloxane/ divinylbenzene (Chartier et al., 2013) vs. polydimethylsiloxane (Szenteczki et al., 2021)]; *in situ* vs. *ex situ* samplings (Scheven, 1994; Marotz-Clausen et al., 2018), and including all vs. only compounds above a specific threshold in relative amounts (Chartier et al., 2013; Szenteczki et al., 2021). Among the 92 compounds chemically identified in this study, more than half (50) were previously unknown to be released by *A. maculatum*. Some of these newly described compounds for *A. maculatum* are known from other species of Araceae (e.g., α-cubebene, β-phellandrene, γ-terpinene, *Sauromatum guttatum*, Hadacek and Weber, 2002), or other plant families (e.g., methyl anthranilate, isobutyl butyrate, citronellal, Knudsen et al., 2006; El-Sayed, 2019). To the best of our knowledge, this study is, however, the first to identify *p*-cresyl butyrate as a floral scent compound.

### Geographic Patterns of Floral Scent

In the region south of the Alps, the qualitative, absolute, and relative differences in scent among populations of *A. maculatum* may be related to the pollinator assemblages that are in this region more diverse in terms of abundance, species composition, and sex ratio (Espíndola et al., 2011; Laina et al., unpublished data). In the region north of the Alps, females of *P. phalaenoides* are the principal pollinators in all studied populations (Espíndola et al., 2011; Laina et al., unpublished data), even though other *Psychoda spp*. also occur in this region (Chartier et al., 2013; Laina et al., unpublished data). Hence, the scent variation we observed among northern populations is not reflected by variations in pollinator spectra in this region. In this study, all variations in scent were more pronounced between regions than among populations within each region. This strong regional component of scent variation in *A. maculatum* across the Alps thus accords with strong differences in pollinator spectra (Espíndola et al., 2011; Laina et al., unpublished data) and coincides with a genetic (AFLP) subdivision of *A. maculatum* across this geographic barrier (Espíndola and Alvarez, 2011). Differing pollinator availability has been linked to different climatic factors (Espíndola et al., 2011), which might also influence scent variation (e.g., Farré-Armengol et al., 2014). However, a preliminary transplant experiment shows that *A. maculatum* individuals originating from north or south of the Alps keep their population-typic scent after transplantation (Gfrerer et al., unpublished data). This suggests that abiotic factors do not directly influence scent emissions (see also Szenteczki et al., 2021). However, we cannot exclude that they might exert differential selection pressures, thus influencing evolutionary processes that may lead to differences in scent emission between the regions. Previous studies in *A. maculatum* also found population effects in scent composition, e.g., Chartier et al. (2013) and Szenteczki et al. (2021). Nonetheless, our study is the first to demonstrate such population differentiation in scent across the Alps. Intraspecific variation in floral scent among populations and regions has also been reported for other plant species (e.g., Dötterl et al., 2005; Chapurlat et al., 2019; and Schlumpberger and Raguso, 2008), including sapromyiophilous species (e.g., Chen et al., 2017). In some of those, this variation, as shown in this study, could be linked to pollinator assemblages and/or genetic patterns (e.g., Chapurlat et al., 2019), but not in others (Dötterl et al., 2005; Schlumpberger and Raguso, 2008).

### Phenotypic Selection on Floral Scents

The two most extensively sampled northern (JOS) and southern (DAO) populations differed in absolute and relative amounts of scent, regardless of whether the analyses were conducted on all compounds, on only those that correlated with relative fruit set and were under selection, or on those that did not correlate with fruit set (Material and methods, Supplementary Figure 3). Thus, this regional difference in scent could be caused by different selection regimes, as well as other reasons, such as phenotypic plasticity (but see Szenteczki et al., 2021) or genetic drift (Herrera et al., 2006; Majetic et al., 2009). In support of differential selection, we detected population-specific signatures of phenotypic selection on scent in JOS and DAO, possibly due to different olfactory preferences of those *Psychoda* species that dominate the pollinator spectra of *A. maculatum* in the northern (female *P. phalaenoides*) vs. southern (and *P. grisescens*) regions (Espíndola et al., 2011; Chartier et al., 2013; Szenteczki et al., 2021; Laina et al., unpublished data).

For the five compounds under phenotypic selection that we were able to chemically identify, i.e., 2-nonanol, 2-heptanol, sabinene, 4-terpinenol and α-terpinene, information on their attractiveness to pollinators of *A. maculatum* is lacking. However, the aliphatic compounds 2-heptanol and 2-nonanol are known, either together or alone, as attractants for bees (Meliponini, Pianaro et al., 2009) and kleptoparasitic flies (Heiduk et al., 2016). They are also known as (sex-)pheromones of female Diptera (Cecidomyiidae, Censier et al., 2014) and female non-Diptera (Trichoptera, Löfstedt et al., 1994). The monoterpenoids sabinene, α-terpinene, and 4-terpinenol are defence substances of some insects (Coleoptera, e.g., Wheeler et al., 2002; Lepidoptera, Ômura et al., 2006) but are used by others (e.g., Lepidoptera, Baur et al., 1993) as oviposition stimulants. The latter two volatiles are also pheromones of fruit flies (Fletcher et al., 1992). In summary, these five compounds, found to be under phenotypic selection, elicit responses in insects other than moth flies. Furthermore, they are known from the floral scent of other sapromyiophilous species (e.g., Hadacek and Weber, 2002; Johnson and Jürgens, 2010), and some of them (α-terpinene and 4-terpinenol) are also known from cattle dung (Dormont et al., 2010; Sládeček et al., 2021), i.e., one of the oviposition substrates of moth flies. Further research is required to establish whether these five compounds, which are all widespread floral scent compounds (Knudsen et al., 2006; El-Sayed, 2019), are attractive to the pollinators of *A. maculatum*.

Several of the compounds most responsible for regional differences in inflorescence scent, e.g., 2-heptanone, 3,7-dimethyloct-1-ene, UNK966 (Supplementary Table 3), did not show signals of phenotypic selection (see Figure 5). Thus, the different selection regimes cannot explain several of the most obvious differences in scent between *A. maculatum* from north and south of the Alps (see also Supplementary Figure 2). However, some other compounds that also differed in absolute amounts between regions (2-heptanol, 2-nonanol, UNK681, sabinene; Supplementary Tables 3, 5) were under phenotypic selection, either in northern JOS or southern DAO (Figure 5), and some of the differences between regions could, therefore, be due to differential selection.

Somewhat unexpectedly, we did not find phenotypic selection for the most abundant compounds in the scent of *A. maculatum*, e.g., indole, β-citronellene, unknown UNK1415, with the exception of 2-heptanol (Figure 5). Even more surprisingly, we also did not find phenotypic selection for those compounds known to attract *P. phalaenoides*, i.e., indole, 2-heptanone, *p*-cresol, and α-humulene (Scheven, 1994; Kite et al., 1998), occurring both north and south of the Alps, and also in JOS and DAO (Espíndola et al., 2011). This contrasts with most other studies, where main compounds and/or pollinator attractants showed signals of phenotypic selection (but see Chapurlat et al., 2019). In *Penstemon digitalis* (Plantaginaceae), one of the main compounds, linalool, was under phenotypic selection and attractive to bumblebees in the laboratory but not in field bioassays (Parachnowitsch et al., 2012; Burdon et al., 2020). Possible explanations for not finding phenotypic selection on the main compounds of *A. maculatum* include the following: (1) their released amounts are high enough to achieve maximum pollinator attractiveness (see also Chapurlat et al., 2019); (2) there are opposing selection pressures on these compounds by different pollinators or herbivores, resulting in zero ‘net' selection (e.g., Knauer and Schiestl, 2017; Chapurlat et al., 2019); and (3) their relationship with flower visitors is non-linear and non-quadratic (e.g., Galen et al., 2011). Although our multivariate models detected non-linear phenotypic selection by including quadratic terms, such quadratic analyses cannot uncover all potential non-linear relationships (e.g., Stinchcombe et al., 2008). Hence, we cannot exclude the possibility that such abundant and/or attractive compounds are still under phenotypic selection, which in turn calls for future statistical developments that allow testing for any kind of non-linear multivariate relationships.

Deceptive plant species might experience stronger selection than rewarding ones (Sletvold and Ågren, 2014). However, by comparison with rewarding species (Parachnowitsch et al., 2012; Gross et al., 2016; Gervasi and Schiestl, 2017; Chapurlat et al., 2019), we found that deceptive *A. maculatum* does not release a higher number of volatiles with signatures of phenotypic selection (7 vs. 3–42%), but these volatiles appear to be under slightly stronger positive linear phenotypic selection (−0.3 to 0.5 vs. −0.3 to 0.4, Min to Max; Figure 5; Parachnowitsch et al., 2012; Gross et al., 2016; Chapurlat et al., 2019) and stronger non-linear phenotypic selection (−0.9 to 9 vs. −0.5 to −0.3, Min to Max; Figure 5; Gervasi and Schiestl, 2017). Future studies on other deceptive plant species that also attract specific pollinators by chemical cues, but have lower levels of fruit set than *A. maculatum* (such as many orchids, e.g., Tremblay et al., 2005), might reveal even stronger signatures of phenotypic selection.

## Conclusions

Our study on sapromyiophilous *A. maculatum* reported the highest number of floral volatiles ever found in a single plant species to date. This chemical hyperdiversity could be due to the fact that *A. maculatum* imitates the odours of a multitude of differently scented breeding substrates of its moth fly pollinators, e.g., dung, fungi, and rotting plant material. We recorded pronounced scent differences between populations from north vs. south of the Alps, and this geographic pattern in scent agrees with previously described pollinator and genetic patterns across this geographic barrier. For the first time, the results of this study provide evidence that floral scents of a deceptive plant are under phenotypic selection and suggest that populational and regional differences in scent are partly due to differential selection, while other reasons such as phenotypic plasticity and genetic drift cannot be excluded. The biological role of most compounds under selection is unknown and awaits determination in future studies in *A. maculatum* and other plants where phenotypic selection on scent was demonstrated (Parachnowitsch et al., 2012; Gross et al., 2016; Gervasi and Schiestl, 2017; Knauer and Schiestl, 2017; Chapurlat et al., 2019).

## Data Availability Statement

The original contributions presented in the study are included in the article/Supplementary Materials, and the R code for the simulation and the full scent dataset can be found in the Dryad Digital Repository (https://doi.org/10.5061/dryad.pnvx0k6kn).

## Author Contributions

SD, MG, AH, and HC designed the research. EG and DL conducted the fieldwork. RF executed the scent sample laboratory work. EG and SD built the scent library. EG analysed all scent and fruit set data, designed and performed the selection analyses, and wrote the first draft of the manuscript. TT identified and synthesised unknown compounds. MH, WT, RF, SD, and EG discussed statistical approaches for selection analyses. MH designed and performed the simulations. All authors contributed to the final version.

## Funding

This study was funded by a grant from the Austrian Science Fund (FWF; P30175-B29) to AH, HC, and SD (PI). All samplings were carried out in compliance with the current laws of the respective countries. MH and WT gratefully acknowledge support from the WISS 2025 project ‘Lab for Intelligent Data Analytics Salzburg’ (20204-WISS/225/197-2019 and 20102-F1901166-KZP).

## Conflict of Interest

The authors declare that the research was conducted in the absence of any commercial or financial relationships that could be construed as a potential conflict of interest.

## Publisher's Note

All claims expressed in this article are solely those of the authors and do not necessarily represent those of their affiliated organizations, or those of the publisher, the editors and the reviewers. Any product that may be evaluated in this article, or claim that may be made by its manufacturer, is not guaranteed or endorsed by the publisher.
